# Development of cisplatin-loaded hydrogels for trans-portal vein chemoembolization in an orthotopic liver cancer mouse model

**DOI:** 10.1080/10717544.2021.1895908

**Published:** 2021-03-09

**Authors:** Xinxiang Yang, Wai-Ho Oscar Yeung, Kel Vin Tan, Tak-Pan Kevin Ng, Li Pang, Jie Zhou, Jinyang Li, Changxian Li, Xiangcheng Li, Chung Mau Lo, Weiyuan John Kao, Kwan Man

**Affiliations:** a Department of Surgery, HKU-SZH and Faculty of Medicine, The University of Hong Kong, Hong Kong, China; b Department of Diagnostic Radiology, Faculty of Medicine, The University of Hong Kong, Hong Kong, China; c Department of Surgery, First Affiliated Hospital of Zhejiang University, Hangzhou, China; d Department of Liver Surgery, First Affiliated Hospital of Nanjing Medical University, Nanjing, China; e Department of Industrial and Manufacturing Systems Engineering, Faculty of Engineering, The University of Hong Kong, Hong Kong, China; f Biomedical Engineering, Faculty of Engineering, The University of Hong Kong, Hong Kong, China; g Department of Chemistry and Chemical Biology Centre, Faculty of Science, The University of Hong Kong, Hong Kong, China; h Li Ka Shing Faculty of Medicine, The University of Hong Kong, Hong Kong, China

**Keywords:** Biomaterial, chemoembolization, caspase 3, hydrogel, liver neoplasms

## Abstract

Transarterial chemoembolization is a standard treatment for intermediate-stage hepatocellular carcinoma (HCC). This study evaluated the anti-tumor effect of the semi-interpenetrating network (IPN) hydrogel as a novel embolic material for trans-portal vein chemoembolization (TPVE) *in vivo*. A nude mice orthotopic HCC model was established, followed by TPVE using IPN hydrogel loaded with or without cisplatin. Portal vein blockade was visualized by MRI and the development of tumor was monitored by IVIS Spectrum Imaging. Tumor proliferation and angiogenesis were evaluated by Ki67 and CD34 staining respectively. Intra-tumor caspase 3, Akt, ERK1/2, and VEGF activation were detected by Western Blot. ^18 ^F-FMISO uptake was evaluated by microPET-MRI scanning. IPN hydrogel first embolized the left branch of portal vein within 24 hours and further integrated into the intra-tumor vessels during 2 weeks after the treatment. Mice treated with cisplatin-loaded hydrogels exhibited a significant decrease in tumor growth, along with lower plasma AFP levels as compared to hydrogel-treated and untreated tumor-bearing mice. By Ki67 and CD34 staining, the TPVE with IPN hydrogel suppressed tumor proliferation and angiogenesis. In addition, increased tumor apoptosis shown by up-regulation of caspase 3 with decreased expressions of tumor cell survival indicators Akt and ERK1/2 were observed in the treatment groups. Consistent with the decreased expression of VEGF after TPVE, hypoxia level in the tumor was also reduced as indicated by ^18 ^F-FMISO uptake level. IPN hydrogel-based TPVE significantly suppressed the tumor development by regulating intra-tumor angiogenesis and cell survival in an orthotopic HCC mouse model, suggesting a viable embolic agent for transarterial chemoembolization.

## 1. Introduction

Primary liver cancer in China accounts for half of the total global cases, in which 70–90% are hepatocellular carcinoma (HCC) (Torre et al., 2016). Transarterial chemoembolization (TACE) remains a standard treatment for intermediate stage HCC and one of the primary choices for downstaging of advanced HCC before liver resection or transplantation (Otto et al., 2013; Sieghart et al., 2015). In conventional TACE, chemotherapeutic agents emulsified with radiopaque Lipiodol are injected through the hepatic artery followed by an embolic particle (Raoul et al., 2019). Chemotherapeutic agents exert a locoregional cytotoxicity against tumor growth and the hepatic artery embolization result in ischemic effects on tumor growth (Golfieri et al., 2011; Lewis & Dreher, 2012). This ability ensures local delivery of chemotherapeutic agents to some extent, thus reducing the undesired systemic side effects and preserving the liver remnant. Lipiodol-based TACE is generally tolerated in most cases, although 4–7% patients undertake major complications (e.g. hepatic abscess, infarction and pulmonary embolism) with near 1% mortality in one month (Liapi & Geschwind, 2011). The survival benefit has been controversial for inconsistent and unstable therapeutic efficacy of TACE (Lencioni et al., 2012; Idee & Guiu, 2013). Such deficit leads to our study to explore the use of biofunctional biomaterials as a novel chemoembolization alternative.

We have established an in-situ photopolymerizable semi-interpenetrating network (IPN) hydrogel system composed of synthetic polymer and biomacromolecules with a wide range of potential applications including local drug delivery (Peppas et al., 2000; Guerra et al., 2017). Our previous study demonstrated that a macrophage-loaded IPN of thiolated gelatin (Gel-PEG-Cys) and polyethylene glycol diacrylate (PEGdA) mimics the extracellular matrix allowing the delivery of cell-derived biomolecules to exhibit anti-cancer functions (Guerra et al., 2017). In recent years, several hydrogels were explored as a potential candidate for embolization material in TACE to increase patient tolerance and to decrease systemic toxicity while providing a depot for local drug delivery (Chan et al., 2018; Chen et al., 2019). For example, Poursaid et al. synthesized a recombinant silk-elastin like protein polymer formulation that can remain liquid at room temperature while transform into solid hydrogels at 37 °C *in vitro* (Poursaid et al., 2015). JS Lym et al. fabricated a pH-sensitive hydrogel specifically forming gel-like structure under low pH condition particularly tumor environment in HCC (Lym et al., 2016). Currently, one of the major challenges remains to be the very limited resident time of the hydrogel in tumor blood vessels due to their active hemodynamics while maintaining the anti-tumor characteristic. In this study, we aimed to optimize and elucidate the anti-tumor capacity and the systemic safety of the robust and biodegradable IPN hydrogel system as a potential embolic agent in TACE.

## 2. Material and methods

### 2.1. IPN hydrogel fabrication

PEGdA/Gel-PEG-Cys hydrogel was synthesized following previously published procedures with some modulations (Burmania et al., 2003; Xu et al., 2012; Guerra et al., 2017). The IPN prepolymer solution was made of 1% (w/v) Iragcure 2959 photoinitiator, 20% w/v PEGdA and 20% w/v Gel-PEG-Cys dissolved in 1× PBS solution (pH 4.0) with or without the addition of cisplatin (1 mg/mL, pH 4.0). Such crosslinked IPN hydrogels exhibited higher maximum weight swelling ratio, higher water content, significantly lower cumulative gelatin dissolution up to 7 days, and lower gel stiffness compared to physically incorporated gelatin hydrogels (Fu et al., 2012; Xu et al., 2013).

We first prepared various formulations (Table 1) of Gel-PEG-Cys plus PEGdA in order to have a quickly formed IPN hydrogel. 10% or 20% w/v PEGdA solution and 10% or 20% w/v Gel-PEG-Cys solution were prepared in pH 4.0 1× PBS at 37 °C. Then Gel-PEG-Cys solution was mixed with PEGdA solution and 1% w/v Iragcure 2959 photoinitiator via vortexing. 200 μL of hydrogel precursor solution was dropped onto the flat glass slide and subjected to long-wavelength UV (λ_max_ = 365 nm, intensity at 100 mW/cm^2^) light until it became a solid gel. As shown in Table 1, higher concentrations of PEGdA solution and Gel-PEG-Cys solution could take a shorter time to form a gel. It took within 2 minutes with 20% w/v PEGdA solution and 20% w/v Gel-PEG-Cys solution, which was fit to the following *in vivo* study.

**Table 1.** Formulation table.

PEGdA (w/v)	Gel-PEG-Cys(w/v)	Iragcure 2959 photoinitiator (w/v)	Time to form a gel
10%	10%	1%	>8 min
10%	20%	1%	4–5 min
20%	10%	1%	4–5 min
20%	20%	1%	<2 min

### 2.2. Mouse ectopic and orthotopic HCC model

The mouse model was established with human MHCC97L cells (Liver Cancer Institute, Fudan University), which were labeled with luciferase according to the protocol as previously described (Guerra et al., 2017). Nude mice (6–8 weeks old, male) purchased from Laboratory of Animal Unit in the University of Hong Kong were used. For establishing nude mice ectopic HCC model, 2 × 10^6^ MHCC97L suspended in 0.1 mL PBS were injected subcutaneously onto the flanks of the mice. The subcutaneous tumor was removed 4 weeks later and used to establish orthotopic HCC model. In brief, the tumor was cut into fragments by surgical scissors and scalpels in DMEM (Gibco, ThermoFisher). The volume of each piece of the tumor was approximately 1 mm^3^. Another group of nude mice were anesthetized to receive tumor implant. The fragment of the tumor tissue was inserted into the left lobe of the liver. The mice with orthotopic tumors were randomly divided into three groups including the negative control group, IPN-treated group, and cisplatin-IPN-treated group. Five mice were included in each group.

### 2.3. Trans-portal vein embolization (TPVE) procedure

The TPVE procedure was modified according to our previous study (Liu et al., 2014). The mice were treated with IPN hydrogels with or without cisplatin at 2 weeks after the tumor fragment was implanted. 20 ul of IPN or cisplatin-loaded IPN hydrogels were injected via main portal vein while the right portal vein was clamped by a mini vessel clamp. The hepatic artery was then ligated. The local injection site was exposed briefly to long-wavelength UV light to ensure gel formation in the left portal vein and in the tumor before the micro-clamp was removed. The peripheral blood, the tumor tissues, and the tumor adjacent liver tissues were sampled at 2 weeks after TPVE treatment. The tumor size was measured with Vernier caliper, and the volume was calculated according to the following formula: V = 0.5 × L × W^2^ (V, volume; L, length; W, width).

### 2.4. Small-animal MR imaging and bioluminescence imaging

Images of the portal vein before and after IPN hydrogel embolization were obtained using a nanoScan^®^ PET–MR imager from Mediso (Mediso kft., Budapest, Hungary). After induction of anesthesia with isoflurane, the mice were positioned in the scanner bed with a heating pad to keep body temperature at 35 °C. During the MR imaging, isoflurane was given at 1.5–3.0% in 500 mL/min medical oxygen, with the attempt to keep the respiratory rate between 55 and 65 bpm. Both T1 and T2 sequences of MRI images were analyzed.

The development of the tumors was detected by IVIS Spectrum *in vivo* imaging system once a week for 4 weeks. Radiance values of the tumor were calculated automatically by the imaging system with a standard circle overlaying the tumor area. The maximum and minimum of scale bar for each bioluminescent image was manually set to the same to avoid signal saturation.

### 2.5. Hematoxylin and eosin (H&E) and immunohistochemistry (IHC) staining

At the end of the treatment period, the animal was sacrificed, and the tumor and the liver samples were removed and fixed in 10% formalin and then embedded in paraffin. Slides (4 μm thick) were deparaffinized in xylene, followed by rinses in ethanol. After rehydration, the slides were treated with hematoxylin and eosin solutions for histological examination. IHC staining was performed to detect the expression of Ki67 and CD34 as described previously (Li et al., 2014).

### 2.6. Tunel assay

DNA fragments (representing apoptosis) was detected by Terminal deoxynucleotidyl transferase dUTP nick end labeling (TUNEL) method. The tissue slides were incubated with 1 µg/ml Proteinase K for 15 min at room temperature, washed twice with PBS, and incubated with TUNEL solution for 60 min at 37 °C in the dark. The slides were then washed with PBS thrice followed by incubation with a chromogenic HRP substrate solution for 30 min at room temperature. The brown color signal generated indicated apoptotic cells.

### 2.7. Serum alpha fetoprotein (AFP) detection

The levels of serum AFP in mice were detected according to the manufacturer’s instruction (Abcam).

### 2.8. Reverse transcription-polymerase chain reaction (RT-PCR)

The expression of intra-tumor VEGF and HIF-1α mRNA were detected by RT-PCR, which was done with a modified protocol as previously described (Li et al., 2014). The human VEGF (sequence forward: GGGTGCAGCCTAAAAGGACC and reverse: AGGGGATGGAGGAAGGTCAA, corresponding to NCBI reference sequence NM_001025366.3) and HIF-1α (sequence forward: TGTAATGCTCCCCTCACCCA and reverse: TGCAGGGTCAGCACTACTTC, corresponding to NCBI reference sequence NM_001530.4) were obtained from Thermo Fisher Scientific Co. Briefly, the PCR reaction mixture was prepared as follows: 2 µl cDNA, 10 µl SYBR Green, 0.1 µl primer mix (10 pmol/µl) and 7.9 µl distilled H_2_O. The PCR running condition was set up as follows: 50 °C 2 mins for 1 cycle, 95 °C 10 mins for 1 cycle, 95 °C 15 seconds with 60 °C 1 min for 40 cycles, 95 °C 15 seconds for 1 cycle, 60 °C 1 min for 1 cycle and 95 °C 15 seconds for 1 cycle. The RT-PCR result was analyzed using ViiA^TM^ 7 software and calculated using 2^^-△△T^ method as relative to that of the normal liver.

### 2.9. Western Blot

Western Blot assay was performed using the SDS-PAGE Electrophoresis System as described previously (Li et al., 2014) with monoclonal antibodies specific for β-Actin (MAB8929, R&D Systems), VEGF (ab46154, Abcam), AKT (9272S, Cell Signaling Technology), phospho-AKT (13038, Cell Signaling Technology), Erk1/2 (4695, Cell Signaling Technology), phospho-Erk1/2 (9101, Cell Signaling Technology), caspase 3 (9662, Cell Signaling Technology) and cleaved caspase 3 (9579, Cell Signaling Technology). Anti-β-Actin antibody was diluted at the concentration of 1:50000 for use. The other antibodies were diluted at 1:2000 as a working concentration.

### 2.10. Statistics analysis

Statistical analysis was conducted using the GraphPad Prism 6 software. Continuous variables were presented as the mean ± standard deviation (SD). Statistical significance of the differences between the experimental groups was determined by Mann-Whitney analysis. *p* value < .05 was statistically significant.

## 3. Results

### 3.1. IPN hydrogel embolized left portal vein and intra-tumor microvessels

**Figure 1.**
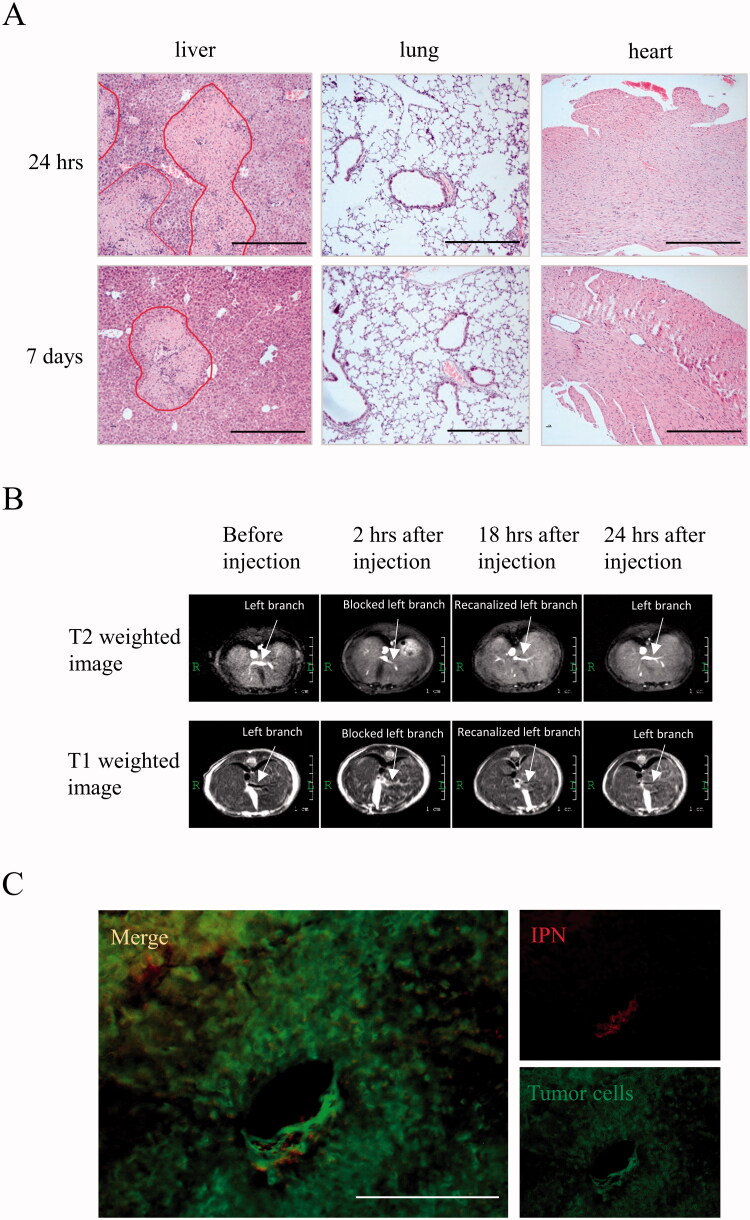
Severe hepatic necrosis in the left lobe of liver was induced by IPN hydrogel injection in early phase. (A) The hepatic histology was detected by H&E staining. Circled areas indicate hepatic necrosis. Bar = 400 μm. (B) The portal vein anatomy was scanned by MRI before and after (at 2, 18, and 24 hours) IPN hydrogel treatment. The upper panel was T2 weighted MRI images and the lower panel was T1 weighted MRI images. (C) The RFP-labeled hydrogels and GFP-labeled tumor cells in the tumor area at 2 weeks after IPN hydrogel treatment were detected by fluorescence microscope. Bar = 200 μm.

After the in situ-photopolymerization of IPN in the left portal vein, severe massive hepatic necrosis in the left lobe of liver was formed (Figure 1(A)). Ischemic injury resulted in periportal and confluent necrosis, where the hepatocytes were replaced by fragments of nucleuses from dead hepatocytes and infiltrated inflammatory cells, involving in multiple hepatic lobules in the left lobe at 24 hours after IPN hydrogel injection (Figure 1(A)). The liver necrosis decreased 7 days later as compared to subacute phase (24 hours) (Figure 1(A)). However, neither inflammation nor necrosis was found in the lung and heart at 24 hours and 7 days (Figure 1(A)). The portal vein anatomy was scanned by MRI at different time points after portal vein embolization. Both T2 weighted and T1 weighted axial MRI images showed that the left branch of portal vein was completely blocked at 2 hours (Figure 1(B)). Afterwards, fading of IPN hydrogels in the left portal vein was observed 24 hours after embolization (Figure 1(B)). These findings indicated that IPN hydrogels blocked the left portal vein in a short term immediately after polymerization and gradually degraded and flowed into the liver to induce severe ischemia injury in the left lobe of the liver.

In the mouse orthotopic HCC model established with GFP-labeled MHCC97L cells, the mice were treated with RFP-labeled IPN hydrogels. The RFP signals appeared in the microvessls in the tumor area two weeks after IPN hydrogel treatment (Figure 1(C)), showing the long-standing residence of the IPN hydrogels in the intra-tumor microvessls.

### 3.2. IPN hydrogel based TPVE suppressed tumor development in an orthotopic HCC mouse model

**Figure 2.**
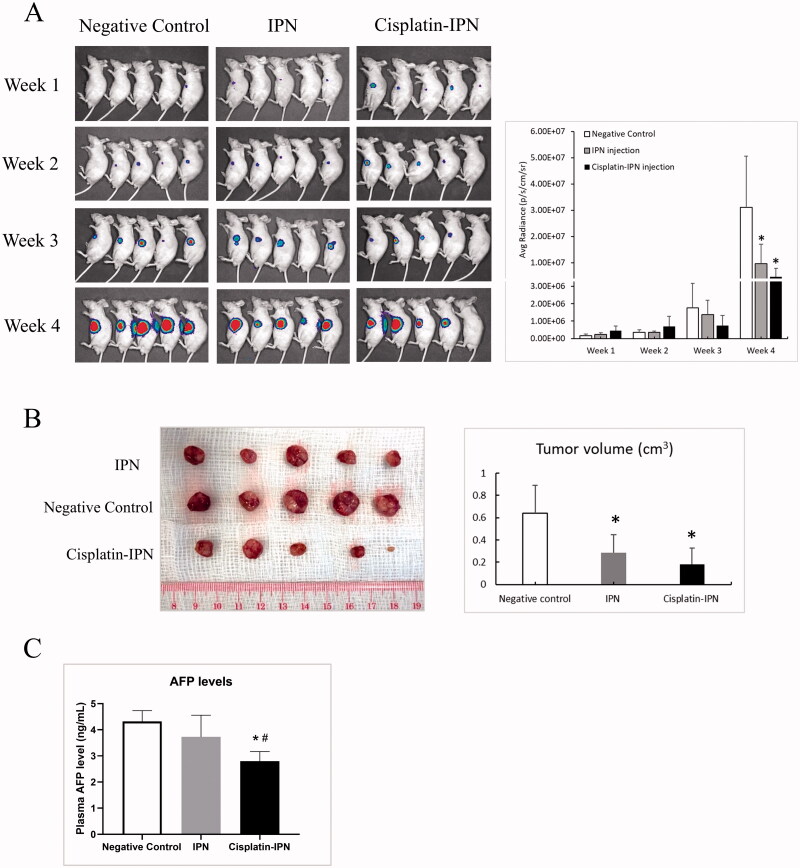
IPN hydrogel based TPVE decreased tumor development (A) IVIS spectrum was introduced to monitor the development of tumor every week after tumor implantation for 4 weeks. The bar chart showed the average radiance signaling (luciferase) of the tumors. (B) Tumor volume was examined and compared from each group. (C) Plasma AFP levels were compared from each group (*N* = 5/group; ∗*p <* .05 differences between treatment group and negative control group; *#p <* .05 differences between treatment groups).

In the mouse orthotopic HCC model established by luciferase-labeled MHCC97L cells (Qi et al., 2016), the mice were divided into three groups: negative control (PBS injection), IPN (treated with IPN hydrogel), and cisplatin-IPN (treated with cisplatin-loaded IPN). IVIS Spectrum Imaging was applied to monitor the development of orthotopic liver tumor. In the first three weeks, there was no significant differences of the luciferase signals among negative control group, IPN group, and cisplatin-IPN group. At week 4, the average levels of luciferase signals from the mice in IPN group (9.61 ± 7.41 × 10^6^, *p* = .050) and cisplatin-IPN group (4.66 ± 3.13 × 10^6^, *p* = .017) were significantly lower than that in negative control group (3.11 ± 1.95 × 10^7^). In addition, the signals in cisplatin-IPN group were lower than that in IPN group, implying an enhanced anti-tumor effect due to the addition of cisplatin (Figure 2(A)). The mice were sacrificed at 4 weeks after tumor implantation. The average volume of the tumors in negative control group were significantly higher than that in IPN and cisplatin-IPN group respectively (0.64 ± 0.25 vs 0.29 ± 0.16 and 0.18 ± 0.15 cm^3^) (Figure 2(B)). The alpha-fetoprotein (AFP) is a specific plasma biomarker for the development of HCC. The AFP level in the mice from cisplatin-IPN group was significantly downregulated than that in negative control group and IPN group respectively (2.797 ± 0.369 vs 4.317 ± 0.414 and 3.727 ± 0.829 ng/mL) (Figure 2(C)). These results indicated that IPN polymerized in TPVE suppressed tumor growth and this effect was further enhanced when IPN hydrogel was loaded with cisplatin.

### 3.3. IPN hydrogel based TPVE suppressed tumor proliferation

**Figure 3.**
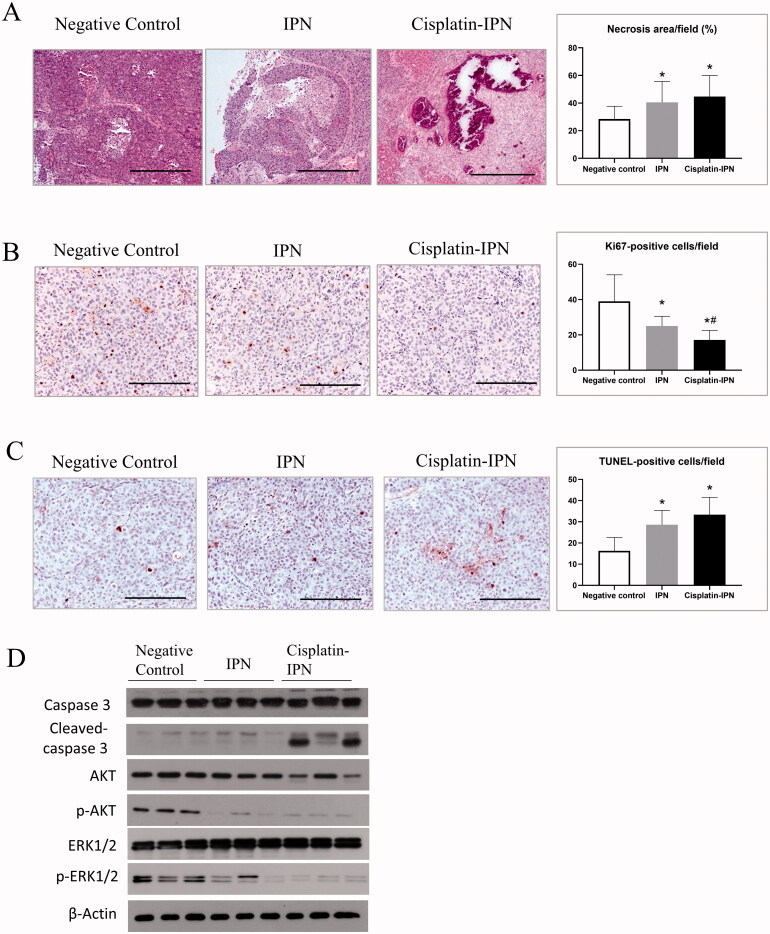
IPN hydrogel based TPVE suppressed tumor proliferation. (A) The tumor histology was detected by H&E staining. Bar = 400 μm. The necrosis area percentages in tumors were compared from each group as shown in the bar chart. (B) The proliferation levels were detected by anti-Ki67 staining. Bar = 200 μm. The Ki67 positive cells per field in the tumors were compared from each group as shown in the bar chart. (C) The apoptosis levels were detected by TUNEL assay. Bar = 200 μm. The TUNEL-positive cells per field in tumors were compared from each group as shown in the bar chart. (D) The expressions of intra-tumor caspase 3, cleaved caspase 3, Akt, phosphorylated Akt, ERK1/2, and phosphorylated ERK1/2 from each group were detected by Western Blot. (∗*p <* .05 differences between treatment group and negative control group; *#p <* .05 differences between treatment groups).

H&E staining of the tumor tissues showed that necrosis areas both in the tumor from IPN group and cisplatin-IPN group were significantly larger than that from negative control group (Figure 3(A)). However, no necrosis or inflammation was observed in adjacent non-tumor areas of each group (Supplementary Figure), indicating that the vessel embolization induced by IPN hydrogels mainly affected tumor microvessels. Ki67 staining was used to evaluate the tumor proliferation ability after TPVE. As expected, the average number of ki67-positive tumor cells was significantly small in IPN group and cisplatin-IPN group compared with that in the negative control group (Figure 3(B)). Moreover, increased impaired tumor proliferation was observed in cisplatin-IPN group than in IPN group due to cisplatin release (Figure 3(B)). TUNEL assay was conducted to examine the apoptosis levels of the tumor in each group. The results showed increased TUNEL-positive cells in IPN group and cisplatin-IPN group compared to the negative control group (Figure 3(C)).

Since caspase 3 is activated generally in the process of cell apoptosis trigged by both extrinsic and intrinsic factors (Ghavami et al., 2009), we further examined its expression level in each group. Western Blot results showed that the cleaved caspase 3 expression increased in both IPN and cisplatin-IPN group, while being more pronounced in the cisplatin-IPN group (Figure 3(D)). On the contrary, the activation of Akt and MAPK/ERK signaling pathways promote cell survival by inhibiting the pro-apoptotic proteins including caspase 3 (Vauzour et al., 2007; Balmanno and Cook, 2009). As shown in Figure 3(D), the result indicated that the phosphorylation of Akt and Erk1/2 expression was down-regulated in IPN and cisplatin-IPN groups. Taken together, the evidences indicated that portal vein embolization and chemoembolization with IPN hydrogel suppressed liver tumor cell proliferation and induced tumor apoptosis.

### 3.4. IPN hydrogel based TPVE restrained tumor angiogenesis and hypoxia

**Figure 4.**
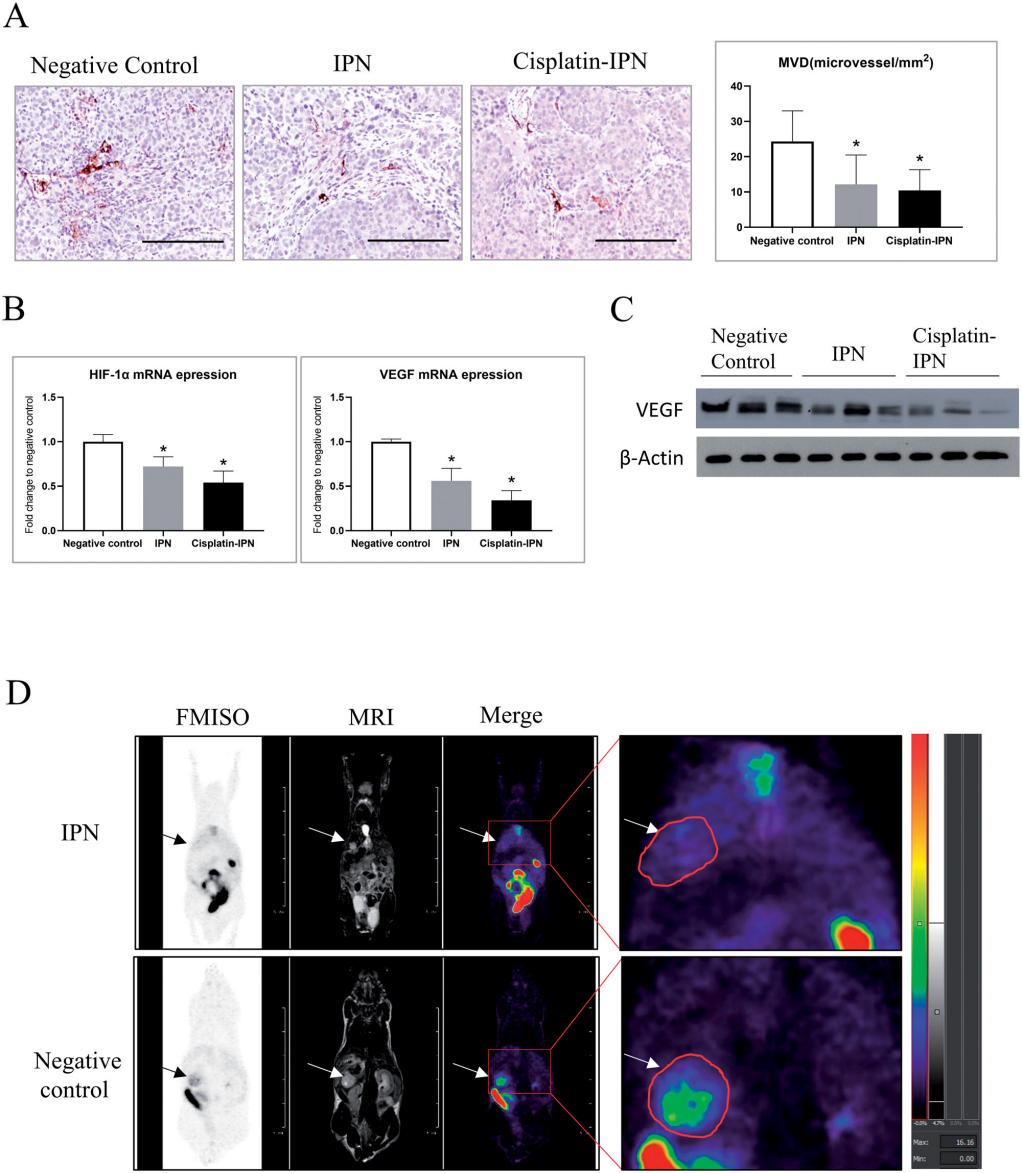
IPN hydrogel based TPVE suppressed tumor angiogenesis. (A) The MVD (microvessel density) was detected by anti-CD34 staining. Bar = 200 μm. The MVD per field in the tumors were compared from each group as shown in the bar chart. (B) The mRNA expression of intra-tumor HIF-1α and VEGFA were detected by RT-PCR. (C) The expressions of VEGF in the tumors from each group was detected by Western Blot. (D) The ^18 ^F-FMISO uptake levels in IPN group and negative control group were captured by microPET-MRI scanning. (∗*p <* .05 differences between treatment group and negative control group).

Tumor microvessel density (MVD) characterized by CD34 expression was assessed to evaluate the degree of angiogenesis in rapidly growing tumor. The results of CD34 staining by IHC showed that the MVD levels both in IPN group and cisplatin-IPN group were notably lower than that from negative control at 4 weeks after tumor implantation (Figure 4(A)), suggesting TPVE treatment down-regulated tumor angiogenesis. Hypoxia-inducible factor 1-alpha (HIF-1α)/vascular endothelial growth factor (VEGF) signaling pathway has been reported to play a key role in generating new blood vessels in response to low oxygen, injury, cytokines and growth factors (Ferrara, 2004; De Francesco et al., 2017). As shown in Figure 4(B), the mRNA expressions of both HIF-1α and VEGF were significantly down-regulated in both IPN and cisplatin-IPN group. As expected, the Western Blot result demonstrated that the intra-tumor expression of VEGF was decreased in IPN group, especially in cisplatin-IPN group, compared to negative controls (Figure 4(C)). Hypoxia is a common phenomenon in the intra-tumor regions of HCC, due to abnormal microvasculature and unrestrained HCC proliferation that leaded to angiogenesis (Xiong et al., 2017). IPN hydrogel blocked the intra-tumor microvessels after TPVE then subsequently reduced oxygen supply to the tumor, which might promote hypoxia in the tumor. Thus, we evaluated the hypoxia levels in the mouse model by detecting ^8 ^F-Fluoromisonidazole (^18 ^F-FMISO), a commonly used PET-MRI tracer for hypoxia, at 4 weeks after tumor implantation. As shown in Figure 4(D), ^18 ^F-FMISO uptake of the tumor in IPN group was significantly lower compared to negative controls, demonstrating a decreased hypoxia after IPN hydrogel treatment.

## 4. Discussion

In the present study, we have fabricated a thiolated Gel-PEG-Cys and PEGdA crosslinked IPN hydrogel loaded with cisplatin, suggesting an embolic particle and drug delivery agent for TACE. This study was the first to report that trans-portal vein embolization using IPN hydrogels demonstrated an anti-tumor effect by decreasing tumor cell survival and angiogenesis. Cisplatin-loaded IPN hydrogels achieved both the blockage of blood supply and the delivery of anti-tumor chemotherapeutics in the orthotopic HCC mouse model.

The efficacy of TACE can be affected by tumor angiogenesis of the residual disease, in which HIF-1α and VEGF play key roles in response to hypoxia (Liang et al., 2010; Liu et al., 2016). Local hypoxia in the tumor induced by TACE can have further downstream effects on the expressions of HIF-1α and VEGF. Studies have shown that both serum HIF-1α and VEGF increased quickly one day after TACE and then decreased gradually to a pre-TACE level (Li et al., 2004; Guo et al., 2012; Ranieri et al., 2015). But the intratumor expression of HIF-1α and VEGF remained controversial. In the present study, the expression of HIF-1α and VEGF in the tumor decreased after both IPN and cisplatin-IPN embolization compared with that in the negative control group. Moreover, CD34 staining in the tumor indicated angiogenesis was suppressed by IPN and cisplatin-IPN treatment. To further identify the tumor hypoxia level after IPN-based TPVE treatment, we performed ^18 ^F-FMISO PET-MRI scanning of the mice. The results revealed hypoxia in the tumor was decreased after IPN-based TPVE treatment.

IPN hydrogels further provided a novel platform to deliver chemotherapeutic drugs in local site. How to increase local concentration of chemotherapeutics has become an active area of research for hydrogel-based delivery. Physical cross-linking of polymer chains can be achieved with a variety of environmental triggers (pH, temperature, ionic strength) and physicochemical interactions (hydrophobic interactions, charge condensation, hydrogen bonding, stereocomplexation, or supramolecular chemistry) (Hoare and Kohane, 2008). In Jae Seung Lym’s study, the authors developed a pH sensitive hydrogel which can form a solid gel in tumor site where the pH is lowered (Lym et al., 2016). As observed in the present study, stronger anti-tumor and pro-apoptosis effects in tumor was induced by cisplatin loaded IPN hydrogel injection compared with pure IPN hydrogels, indicating the feasibility of IPN hydrogels as a drug-delivery platform. After cisplatin-IPN based TPVE treatment, we observed the necrosis in tumor sites but not in tumor adjacent liver tissues, suggesting cisplatin-IPN embolization mainly affected the local tumor site but not the normal liver tissue. Cisplatin is a well-established chemotherapeutic agent in the treatment of various human cancers including lung (Pietanza et al., 2016), bladder (Kamat et al., 2016), ovarian (Armstrong et al., 2006), and liver cancer (the Liver Cancer Study Group of Japan, 2013). Hydrogel-based delivery can provide prolonged drug release and achieve local delivery (Narayanaswamy and Torchilin, 2019). In our mouse model, the IPN hydrogels blocked the left portal vein in a short time while resided in the tumor microvessls till the experimental endpoint. The release speed of solutes by such IPN hydrogel can be controlled depending on the composition of gelatin and PEG (Fu and Kao, 2010; Guerra et al., 2017). One of the limitations in our study was that the release speed of cisplatin was unknown after TPVE. We speculated the cisplatin release on the facts of enhanced anti-tumor effects.

## 5. Conclusions

In summary, our study demonstrated the therapeutic effects of IPN hydrogel based trans-portal vein chemoembolization for tumor treatment in an orthotopic HCC mouse model. IPN hydrogel based TPVE suppressed the tumor progression through down-regulating cell survival and intra-tumor angiogenesis. In addition, IPN hydrogel loaded with cisplatin further displayed its capability in local chemotherapeutic drug delivery to the tumor site.

## Supplementary Material

Supplemental Material
