# Supplementary figures and images for: Development of cisplatin-loaded hydrogels for trans-portal vein chemoembolization in an orthotopic liver cancer mouse model
Source: Drug Deliv. 2021 Mar 9;28(1):520–9. doi: 10.1080/10717544.2021.1895908 (PMC7946021; doi:10.1080/10717544.2021.1895908)

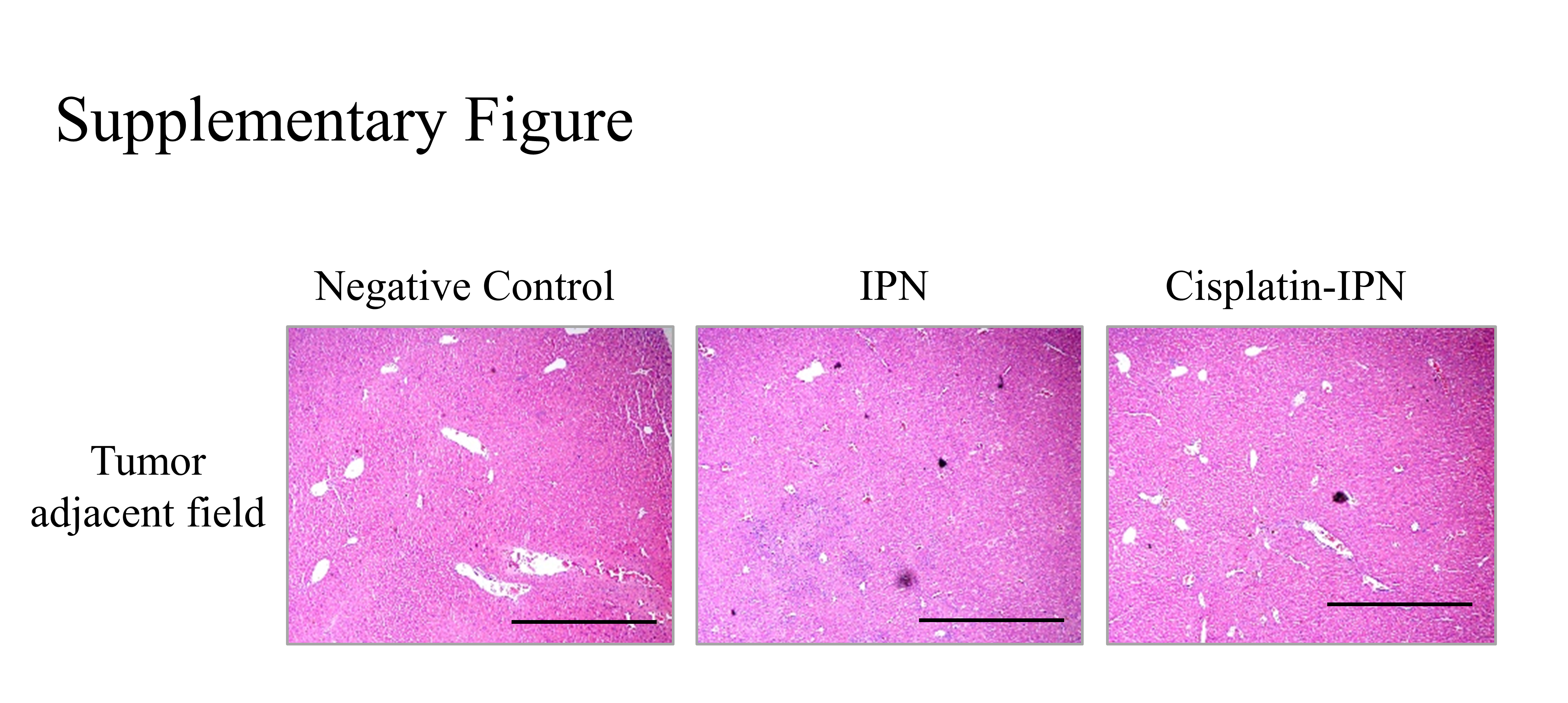

Supplement: Supplemental Material [file IDRD_A_1895908_SM5499.tif]
